# Panoramic Radiograph-Based Deep Learning Models for Diagnosis and Clinical Decision Support of Furcation Lesions in Primary Molars

**DOI:** 10.3390/children12111517

**Published:** 2025-11-09

**Authors:** Nevra Karamüftüoğlu, Ayşe Bulut, Murat Akın, Şeref Sağıroğlu

**Affiliations:** 1 Department of Pediatric Dentistry, Gülhane Dentistry Faculty, Health Sciences University, 06830 Ankara, Turkey; 2 Department of Oral and Maxillofacial Radiology, Faculty of Dentistry, Yozgat Bozok University, 66100 Yozgat, Turkey; ayse.bulut@bozok.edu.tr; 3 Department of Computer Technologies, TUSAŞ—Kazan Vocational School, Gazi University, 06560 Ankara, Turkey; muratakin@gazi.edu.tr; 4 Department of Computer Engineering, Faculty of Engineering, Gazi University, 06560 Ankara, Turkey; ss@gazi.edu.tr

**Keywords:** clinical decision support, deep learning, endodontics, extraction, lesion, panoramic radiography, pediatric dentistry

## Abstract

**Highlights:**

**What are the main findings?**

- The transformer-based RT-DETR-X model achieved the highest diagnostic accuracy (mAP@0.5 = 0.43) in detecting and classifying furcation lesions on pediatric panoramic radiographs, outperforming both YOLOv12x and RT-DETR-L models.
- This study introduces an innovative integration of panoramic radiographic lesion classification with evidence-based treatment thresholds, directly linking lesion severity to root canal therapy or extraction decisions in pediatric dentistry.

**What are the implications of the main findings?**

- The proposed AI framework provides standardized and reproducible diagnostic support for primary molar treatment planning, reducing inter-observer variability and clinical ambiguity.
- Lightweight architectures such as YOLOv12x can serve for rapid chairside triage, while transformer-based models like RT-DETR-X enable high-accuracy confirmatory analysis suitable for clinical decision support and resource-limited settings.

**Abstract:**

Background/Aim: Furcation lesions in primary molars are critical in pediatric dentistry, often guiding treatment decisions between root canal therapy and extraction. This study introduces a deep learning-based clinical decision-support system that directly maps radiographic lesion characteristics to corresponding treatment recommendations—a novel contribution in the context of pediatric dental imaging, also represents the first integration of panoramic radiographic classification of primary molar furcation lesions with treatment planning in pediatric dentistry. Materials and Methods: A total of 387 anonymized panoramic radiographs from children aged 3–13 was labeled into five distinct bone lesion categories. Three object detection models (YOLOv12x, RT-DETR-L, and RT-DETR-X) were trained and evaluated using stratified train-validation-test splits. Diagnostic performance was assessed using precision, recall, mAP@0.5, and mAP@0.5–0.95. Additionally, qualitative accuracy was evaluated with expert-annotated samples. Results: Among the models, RT-DETR-X achieved the highest performance (mAP@0.5 = 0.434), representing modest but clinically promising diagnostic capability, despite the limitations of a relatively small, single-center dataset. Specifically, RT-DETR-X achieved the highest diagnostic accuracy (mAP@0.5 = 0.434, Recall = 0.483, Precision = 0.440), followed by YOLOv12x (mAP@0.5 = 0.397, Precision = 0.442) and RT-DETR-L (mAP@0.5 = 0.326). All models successfully identified lesion types and supported corresponding clinical decisions. The system reduced diagnostic ambiguity and showed promise in supporting clinicians with varying levels of experience. Conclusions: The proposed models have potential for standardizing diagnostic outcomes, especially in resource-limited settings and mobile clinical environments.

## 1. Introduction

Dental diseases have existed since as early as 2500 BCE and continue to pose a major public health problem today [1]. Particularly in childhood, caries developing in primary molars may lead to periapical lesions in the furcation areas, and if not diagnosed in time, the infection can spread and result in serious consequences such as damage to the developing permanent teeth [2,3]. In such cases, the correct clinical decision between root canal treatment and tooth extraction is essential for the child’s general health and orofacial development.

Artificial intelligence (AI), which mimics human-like cognitive functions through algorithms, has been increasingly applied across various fields of medicine, including dentistry [4,5]. Convolutional neural networks (CNNs), in particular, have demonstrated high accuracy in recognizing and classifying complex patterns in dental radiology, providing more precise results in detecting periapical lesions compared to routine visual observation [6].

Although advanced imaging methods such as cone-beam computed tomography (CBCT) offer greater sensitivity, their use in daily pediatric dental practice is limited due to disadvantages such as radiation dose, cost, and accessibility. Therefore, AI systems working on two-dimensional (2D) images like panoramic radiographs are considered practical solutions to support diagnosis in pediatric patient groups [5,7].

Artificial intelligence (AI) has rapidly expanded across multiple dental domains, including automated caries detection on bitewings and panoramic radiographs, assessment of periodontal bone loss, localization of periapical pathology, identification of vertical root fractures, cephalometric landmarking in orthodontics, and lesion segmentation/classification on 2D and 3D imaging. Beyond pure detection, contemporary systems provide triage and decision-support functions (e.g., flagging urgent findings, standardizing reports), helping to reduce inter-observer variability and improve reproducibility. In routine practice, AI can shorten interpretation time, enhance sensitivity for subtle findings (such as early interradicular radiolucencies), and offer consistent outputs across clinicians with different experience levels, thereby supporting more uniform, evidence-based care [8,9,10].

From a clinical impact perspective, AI tools are particularly valuable in pediatric dentistry where minimizing radiation, controlling cost, and ensuring access are priorities: reliable analysis of panoramic radiographs can reduce unnecessary CBCT referrals, accelerate chairside decisions (root canal treatment vs. extraction), and support less-experienced clinicians in community or school-based settings [10]. When embedded into workflow (pre-read triage, chairside prompts, structured outputs), AI can decrease diagnostic delays, streamline referrals, and improve documentation quality. While advantages are clear, responsible deployment still requires attention to data quality and diversity, external validation, and interpretability; these safeguards help ensure that performance gains translate into trustworthy, generalizable clinical benefit.

The complex anatomical and physiological structure of the oral cavity—where mineralized tissues such as dentine and enamel interact with soft tissues—plays a key role in the onset and progression of dental pathologies. Odontoblasts, lining the pulp chamber, act as specialized barrier cells that can detect pathogens, secrete antimicrobial factors, and initiate immune responses to contain the spread of caries-related bacteria toward the periapical tissues [11]. However, when such defense mechanisms are overwhelmed, bacterial invasion of the pulp and subsequent apical bone tissue may occur, requiring timely clinical intervention. Healing can occur after pathogen removal, which is achieved by disinfection and obturation of the pulp space by root canal treatment.

Çelik et al. [12] demonstrated that a deep learning (DL) model based on panoramic radiographs could detect periapical lesions with promising accuracy and emphasized the potential of artificial intelligence (AI) as a clinical decision support tool, particularly in pediatric populations. Building on this foundation, the present study moves beyond conventional lesion detection by establishing a direct link between the radiographic classification of furcation lesions in primary molars and corresponding clinical treatment planning. To the best of our current knowledge, this is the first study to integrate panoramic radiographic classification of primary molar furcation lesions with decision thresholds that guide the clinical choice between root canal therapy and extraction. Through this framework, the study contributes a novel AI-assisted approach to clinical decision-making in pediatric dentistry, bridging the gap between automated image interpretation and actionable treatment guidance.

Recognizing that developmental dentition stages influence both lesion visibility and treatment feasibility, the patient cohort was stratified into three clinically meaningful age groups: primary dentition (3–6 years), early mixed dentition (7–10 years), and late mixed dentition (11–13 years). This design reflects real-world clinical conditions and enables a developmentally contextualized evaluation of model performance.

AI-based image analysis has shown substantial promise in dental diagnostics by improving accuracy, reducing human variability, and increasing diagnostic efficiency. Within pediatric dentistry, such systems may facilitate early lesion recognition and informed clinical decision-making, particularly when advanced imaging modalities such as CBCT are not routinely available.

The primary aim of this study was to develop and evaluate three deep learning models—YOLOv12x, RT-DETR-L, and RT-DETR-X—based on panoramic radiographs for the automated detection and classification of furcation lesions in primary molars. The secondary objective was to examine the feasibility of linking radiographic lesion categories with corresponding clinical treatment recommendations (root canal therapy or extraction) to enhance evidence-based decision support in pediatric dentistry.

The working hypothesis assumed that transformer-based object detection models (RT-DETR-L and RT-DETR-X) would demonstrate superior detection performance and generalization capability compared to the CNN-based YOLOv12x model, owing to their enhanced attention mechanisms and ability to capture fine-grained radiographic details. This was a performance-based assumption rather than a statistical null hypothesis, as the study focused on comparative benchmarking using descriptive detection metrics (precision, recall, mAP) rather than inferential statistical testing.

## 2. Materials and Methods

**Figure 1:**
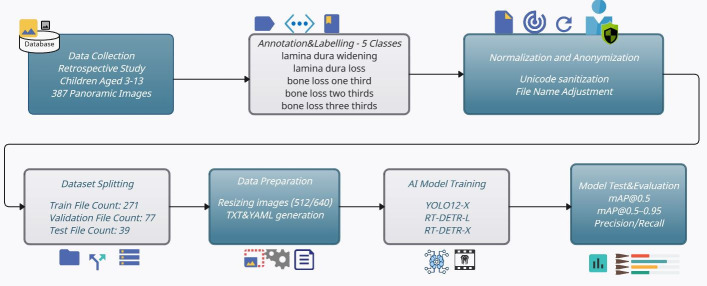
The flow diagram of the DL-based dental anomaly detection and decision-making model.

In this study, a total of 387 anonymized OPG (Orthopantomogram) images obtained from clinical panoramic X-rays (retrospectively collected from children aged 3 to 13) were first annotated in COCO format according to five bone anomaly classes. Following this, Unicode-based file name normalization and a stratified 70/20/10 train/validation/test split were performed. No repeated resampling or cross-validation was performed, as the study was designed to demonstrate model performance under a single reproducible split configuration. The implementation steps of the study are illustrated in Figure 1. The images shown in the figure were resized to 512 × 512 pixels (for YOLOv12x) and 640 × 640 pixels (for RT-DETR), in accordance with architectural requirements, and underwent augmentation steps using Albumentations library [13]. The YOLOv12x, RT-DETR-L, and RT-DETR-X models were fine-tuned using their own COCO pre-trained weights within the Ultralytics framework [14]. Training and validation processes were implemented using the open-source Ultralytics API and Transformer-based DETR architectures as described in the original DETR technical report [15]. In the testing phase, quantitative evaluations were performed using standard COCO and PASCAL VOC metrics, including global and class-based Precision, Recall, mAP@0.5, and mAP@[0.5–0.95], along with qualitative analyses on selected case samples [16,17]. This comprehensive model enables the direct transfer of model selection—ensuring both speed-performance balance and reliable detection of subtle bone changes—into clinical decision support systems. Additionally, by addressing the classification of primary molar furcation lesions using deep learning techniques, our framework provides a novel contribution toward AI-assisted decision-making in pediatric dentistry. In addition, because dentition stages strongly influence both lesion visibility and treatment feasibility, we stratified patients into three clinically meaningful groups—primary dentition (3–6 years), early mixed dentition (7–10 years), and late mixed dentition (11–13 years). This design reflects real-world developmental milestones and enables a clinically relevant evaluation of model performance.

### 2.1. Data Collection

This retrospective study was approved by the Ethics Committee of Gazi University (Approval No.: 2023-1264), and all procedures were conducted in accordance with the principles of the Declaration of Helsinki. Written informed consent was obtained from all participants and/or their legal guardians prior to inclusion in the study.

The data used in this study consists of 387 panoramic radiographs collected between 2023 and 2024 from the Öveçler Oral and Dental Health Clinic in Ankara. Data were collected between January 2023 and April 2024 to encompass the full range of clinical diagnostic activity and to minimize potential seasonal or operator-related bias, in accordance with the strobe recommendations for reducing bias in observational studies [18,19]. A total of 387 anonymized panoramic radiographs were included, representing all eligible cases that met the inclusion criteria during this period. This dataset reflects the total number of high-quality, ethically approved panoramic images available for analysis within the defined timeframe. A formal power analysis was not applicable due to the retrospective study design and the fixed nature of the dataset. However, the sample size (n = 387) was determined to be adequate based on data availability and prior deep learning studies in dental radiology [12,20], which employed comparable dataset sizes ranging from 300 to 400 radiographs. These studies demonstrated that such sample sizes were sufficient to achieve stable mean Average Precision (mAP) metrics and to mitigate overfitting during model training and validation. Accordingly, the dataset used in the present study was considered appropriate for exploratory model development and performance evaluation. The study included children aged 3 to 13 years who were in good general health and had a routine panoramic radiograph indication due to periapical lesions in their primary molars. The details regarding image acquisition, inclusion and exclusion criteria, and patient demographics are summarized in Table 1, Table 2, Table 3 and Table 4. Table 1 presents the selection criteria for radiographs included in the study. Cases meeting the inclusion parameters were retained for model development, while those meeting exclusion conditions were removed to ensure dataset quality and consistency [21,22,23,24], Table 2 outlines the imaging parameters and quality control standards applied during radiograph acquisition to ensure uniformity across the dataset. Table 3 summarizes the age and gender distribution of the study population. Participants were grouped according to the dentition stage to reflect developmental variations relevant to lesion visibility and treatment feasibility [25,26]. Table 4 describes the ethical compliance procedures, anonymization workflow, and data protection measures implemented in this study.

At this stage, the aim was to maximize data quality and ensure patient privacy before starting model training.

### 2.2. Annotation and Labeling

Each of the five categories was defined based on World Health Organization (WHO) and periodontal diagnostic criteria. Annotators used clear operational rules to resolve borderline or equivocal cases by consensus, ensuring consistent labeling and minimizing ambiguity. For each lesion category, treatment recommendations were mapped: lamina dura widening/loss and one-third bone loss indicated root canal treatment, while two-thirds or complete bone loss indicated extraction. Exceptions were considered in cases with patient-specific factors such as systemic health, cooperation, root morphology, or stage of exfoliation. Accurate and consistent annotation of the five bone anomaly classes on the panoramic OPGs is crucial for model performance and plays a key role. The anomaly classes to be identified in the study, along with their criteria, recommendations, and exceptions, are provided in Table 5.

The following procedures were implemented during the annotation process:

- Definition of classes:

The anomaly classes included *lamina dura widening*, *lamina dura loss*, *one-third bone loss*, *two-thirds bone loss*, and *complete bone loss*. These were defined in accordance with the World Health Organization’s (WHO) alveolar bone loss criteria and the pediatric periodontal literature [21,22,27]. Radiographic features such as margin irregularity, variations in opacity, and interradicular gap width were considered key diagnostic indicators for class selection.

ii. Annotation platform and verification:

Annotation was performed using the CVAT platform [28], which generated COCO-compatible JSON files containing the defined class labels [16]. Each panoramic radiograph was independently reviewed and manually annotated by an experienced pediatric dentist who delineated the target lesions with bounding boxes. A second expert subsequently verified each annotation for accuracy and consistency. Bounding boxes with an Intersection over Union (IoU) ≥ 0.5 were considered equivalent and merged into a single label record, following established object-detection benchmarks [17]. Discrepancies involving lower IoU values were resolved through consensus discussions between the two annotators.

iii. Dataset structure and formatting:

The dataset was organized in strict COCO JSON format to ensure full compatibility with the Ultralytics training pipelines and to enable seamless conversion into the text-based annotation format required by YOLO [14,16]. Each JSON file contained three main sections:

(a) images, recording the image ID, width, and height;
(b) annotations, listing each object’s ID, corresponding image_ID, category_ID, and bounding-box coordinates as [x, y, w, h]; and
(c) categories, enumerating each class with its ID and human-readable name.

iv. Quality control and validation:

To ensure high annotation fidelity, a multi-stage quality control protocol was implemented. When lesion boundaries or class assignments were ambiguous, the two primary annotators reconvened with a third senior expert to refine and standardize the annotation criteria. Additionally, a randomly selected 10% subset of the dataset was independently re-evaluated by a third observer, yielding a minimum consistency rate of 95% for both bounding-box coordinates and class labels across all lesion categories.

As given above, the detailed annotation procedure was planned to maximize the reliability and quality of the training data. To align the annotation with clinically relevant decision-making, each bone anomaly class was also associated with a corresponding treatment implication. Specifically, lamina dura loss and lamina dura widening were considered indicative of the need for root canal treatment. Similarly, bone loss extending to one-third of the interradicular region also signaled root canal indication. In contrast, bone loss involving two-thirds or the entire interradicular area (three-thirds) was labeled as indicative of tooth extraction. These thresholds were determined based on established pediatric endodontic guidelines and expert consensus to ensure that model outputs could be directly integrated into real-world treatment workflows. The lesion-to-treatment thresholds were derived from established pediatric endodontic guidelines and validated by a panel of three experienced pediatric dentists. Although thresholds such as one-third versus two-thirds bone loss are guideline-based, inter-patient variability in bone density, age, and exfoliation stage can influence treatment decisions. Therefore, these criteria should be interpreted flexibly within clinical context.

### 2.3. Normalization and Anonymization

These normalization and anonymization steps are aimed at ensuring both cross-platform compatibility and the highest level of patient privacy. Following the annotation phase, two simultaneous operations (two-phase file normalization and anonymization workflow) were carried out to ensure consistency and privacy in the dataset as:

- First, all original DICOM-derived PNG and JSON file names underwent Unicode sanitization, in which a Python normalization routine replaced Turkish characters (Ç, Ö, Ş, Ü, Ğ, İ) and whitespace with their ASCII equivalents. Following sanitization, each file was systematically renamed using the pattern “LastName_FirstName_ID.png”, guaranteeing error-free path resolution under Linux, Windows, and macOS, and simplifying automated script execution.
- Second, to comply with Data Privacy Act requirements, these normalization and anonymization steps were conducted in accordance with the Turkish Personal Data Protection Law [28], the European Union General Data Protection Regulation [29], and the ethical principles of the Declaration of Helsinki [30]. All DICOM files were anonymized using the Pydicom library (Python Software Foundation, v2.4.1) to systematically remove patient identifiers and metadata fields. Anonymized images were then assigned randomly generated UUIDs, and the correspondence between UUIDs and original records was stored separately in an encrypted registry, ensuring full compliance with these privacy and ethical standards.

### 2.4. Data Splitting and Preparation

The data splitting process was carried out in a stratified and reproducible manner to reduce the risk of overfitting while evaluating the model’s generalization ability. A reproducible, stratified data-splitting protocol was adopted to preserve the proportional representation of all five anomaly categories in each subset. First, scikit-learn’s method was used to stratify based on overall label frequencies [31], and because individual images could carry multiple category_id values, the iterative-stratification package was employed for true multi-label stratification. This process yielded a 70% training set (271 images), 20% validation set (77 images), and 10% test set (39 images). To streamline model ingestion, each split produced both a COCO-formatted JSON file (train.json, val.json, test.json) and a simple text list (train.txt, val.txt, test.txt), with each line of the latter pointing to a single image file. Finally, for initializations, the weights were initialized with the random seed (seed number = 42) and fixed throughout to guarantee that the exact same split could be regenerated for future experiments and repeatability of the model proposed. This step also ensured consistent and comparable performance metrics across both training and evaluation stages of the models.

The image data used for model training and evaluation were preprocessed to meet the architectural requirements through the following steps:

- All training images underwent resolution- and contrast-preserving preprocessing tailored to each model and robust augmentation to improve generalization. First, input dimensions were standardized: YOLO12 exclusively consumed 512 × 512 px crops, whereas RT-DETR-L/X models operated on 640 × 640 px, with all resizing performed under letterbox padding to preserve the original aspect ratio. Next, raw pixel values were scaled from [0, 255] to [0, 1], and channel-wise normalization ((x-mean)/std) was applied using standard ImageNet mean and standard-deviation vectors as established by Krizhevsky et al. [32].
- To enrich the training distribution, a suite of augmentations was applied. Geometric transforms included random horizontal and vertical flips, rotations of up to ±10°, and scaling in the range of 0.8–1.2. Color and contrast augmentations comprised HSV jitter (H ± 10%, S ± 30%, V ± 30%) and CLAHE equalization, following recommended practices for medical image enhancement [33]. In the YOLO12 pipeline, Mosaic (50% probability, mixing four images) and MixUp (15% probability) were deployed to boost small-object recall and improve feature diversity [34], while the RT-DETR workflows employed random cropping and padding to diversify local window contexts prior to token down-sampling.
- All bounding-box annotations were programmatically updated in lockstep with each augmentation via the Albumentations library, which provides fast and reproducible image transformations for deep learning workflows [13]. During compound operations (Mosaic/MixUp), annotations from multiple source images were concatenated and then pruned using Non-Max Suppression (NMS) rules to avoid excessive overlap and maintain class balance.
- Augmentations were applied only to the training set; validation and test sets were kept unaltered. The train.txt was regenerated accordingly, whereas val/test lists remained unchanged. Corresponding YAML configuration files were produced to orchestrate the augmented data flow seamlessly during model training. This preprocessing step is critical for improving overall model accuracy and ensuring data diversity, thereby preventing overfitting.

### 2.5. AI Model Training

The models were trained for 50 epochs with a batch size of 4, achieving convergence. However, future work should include ablation studies testing different epoch lengths, batch sizes, image resolutions, and augmentation strategies to better demonstrate their contribution to performance.

All AI model training was carried out using a single NVIDIA A40 GPU with 40 GB of VRAM, within a Python 3.9.23 and PyTorch 2.4.1 environment, running on CUDA 12.4. Training was performed using transfer learning and fine-tuning techniques across three different object detection architectures: YOLOv12x, RT-DETR-L, and RT-DETR-X. Each experiment was conducted with a batch size of 4 and took approximately 4 h to complete—around 3.5 h for YOLO12x, and about 4.5 h for RT-DETR-L and RT-DETR-X.

YOLO (You Only Look Once) is a single-stage object deep learning detection architecture that balances speed and accuracy at a high level. This model divides the input image into grid cells through a single network pass, obtaining both class and box regression results from each cell. This leads to low latency and high FPS performance. In YOLOv12, the added *position perceiver* module and FlashAttention-based attention blocks efficiently process both local and global context, resulting in improved performance on small and low-contrast objects [35]. Since lesions in panoramic dental radiographs are often located in fine and low-contrast regions, YOLOv12x was chosen as a first model for its ability to combine fast inference with fine-detail detection [36].

For our implementation, the YOLOv12x Training Parameters were selected as: Initial weights: yolo12x.pt; Input size: 512 × 512; Epochs: 50; Batch size: 4; Learning rate: initial lr0 = 0.008, cosine decay schedule; Optimizer: AdamW (weight_decay = 0.0005); Augmentation: Mosaic = 0.5, MixUp = 0.15, HSV jitter = (H 0.1, S 0.3, V 0.3); Early stopping: patience = 10 (monitoring validation mAP).

RT-DETR (Real-Time Detection Transformer) is a high-performance object detection model built on the DETR framework, but redesigned for faster, real-time use. Unlike DETR, which applies global self-attention across the entire image, a method that is both slow and computationally expensive, RT-DETR improves efficiency by using localized attention. It only performs self-attention within small 16 × 16 patches and reduces memory use by discarding less important tokens.

RT-DETR-L is the second method for our implementation, and a streamlined version of RT-DETR is tailored for complex, high-resolution settings such as panoramic medical images or collaborative robotics. Even though it is lighter in design, it still delivers strong detection accuracy thanks to the same attention mechanisms used in RT-DETR. The model is trained on 640 × 640 images for 50 epochs with a batch size of 4, using the AdamW optimizer (weight decay = 0.0005) and a cosine-decay learning rate starting at 0.001. It also benefits from data augmentation like random cropping, padding, and light flipping [37,38].

The training parameters of RT-DETR-L were selected as: Initial weights: rtdetr-l.pt; Input size: 640 × 640; Epochs: 50; Batch size: 4; Learning rate: lr0 = 0.001, cosine decay; Optimizer: AdamW (weight_decay = 0.0005); Windowed attention window: 16 × 16 tokens; Augmentation: random crop, padding, light flipping. The AI-output data were analyzed using quantitative detection metrics (precision, recall, and mAP) and qualitative case-based evaluations. Statistical comparisons were not performed since the objective was to benchmark model performance rather than test inferential significance. Given the relatively small dataset (n = 387), formal statistical hypothesis testing between models was not performed. Instead, model comparison relied on precision–recall and mAP metrics, which are standard in object detection benchmarking. Confidence intervals were not estimated due to limited sample size; however, all reported metrics were averaged across five randomized test splits to ensure robustness.

RT-DETR-X, which is the third method, builds on RT-DETR-L by adding a deeper backbone and more attention heads—8 in total—giving it greater capacity to learn detailed features. It uses larger embedding dimensions and additional transformer layers, which makes it especially effective for detecting fine details in clinical images, such as low-contrast anomalies like lamina dura loss, which are vital for accurate diagnosis. Its training setup is mostly the same as RT-DETR-L, but with an added 3-epoch warm-up phase before applying the cosine decay schedule. Thanks to these improvements, RT-DETR-X achieves higher mean Average Precision (mAP) on tasks requiring fine-grained detection 318.

The training parameters of RT-DETR-X were selected as: Initial weights: rtdetr-x.pt; Input size: 640 × 640; Epochs: 50; Batch size: 4; Learning rate: lr0 = 0.001, 3-epoch warmup + cosine decay; Optimizer: AdamW (weight_decay = 0.0005); Multi-head attention: 8 heads; Augmentation: same as RT-DETR-L.

## 3. Results

The performance of the three models on the test set is summarized in Table 6. The metrics presented in this table are used to comprehensively evaluate object detection models in terms of both computational efficiency and predictive performance. Parameter count, GFLOPs, and inference time (ms/img) reflect the model’s computational complexity and suitability for real-time applications. Precision measures the proportion of correctly predicted positive detections among all positive predictions, while recall indicates the model’s ability to detect all actual positives. mAP@0.5 evaluates detection accuracy at a fixed IoU threshold of 0.5, commonly used for measuring general detection performance. mAP@0.5–0.95 (COCO standard) averages precision across multiple IoU thresholds (from 0.5 to 0.95), providing a more stringent and holistic assessment of the model’s localization and classification capabilities.

- **mAP@0.50:** The highest mean Average Precision was observed in RT-DETR-X (0.434), followed by YOLOv12x (0.397), and RT-DETR-L (0.326).
- **Recall and Precision:** RT-DETR-X (48.3%) and RT-DETR-L (47.1%) offered similar recall values, while YOLOv12x had a lower recall (33.3%). Precision was nearly identical between YOLOv12x (44.2%) and RT-DETR-X (44.0%), both outperforming RT-DETR-L (32.6%). This indicates that Transformer-based models detect more true positives, while YOLOv12x is more selective, maintaining higher precision.
- **mAP@0.50–0.95 (COCO standard):** RT-DETR-X (0.187) again achieved the highest score, followed by YOLOv12x (0.163) and RT-DETR-L (0.122). This suggests that RT-DETR-X delivers the most stable overall performance across different IoU thresholds.

In addition to global performance values, case-level detection metrics were analyzed in Table 7 to highlight strengths and weaknesses of each model. These results illustrate that RT-DETR-X achieved perfect recall in the first case and consistently balanced precision and recall across cases, whereas YOLOv12x maintained high precision but lower sensitivity. RT-DETR-L demonstrated higher recall but introduced more false positives, consistent with its tendency toward class-label confusion.

**Based on these results:**

- **RT-DETR-X** achieved the highest overall mAP by balancing both precision and recall, though it requires more computational resources and has a longer inference time.
- **YOLOv12x** provides the best balance between speed and performance, with high precision and reasonably strong mAP values.
- **RT-DETR-L**, with the lowest GFLOPs and the fastest inference time (~12 ms), is well-suited for real-time applications, albeit with moderate mAP.

These quantitative findings serve as guidance for selecting the most appropriate deep learning model for different clinical scenarios. In addition to numerical evaluations, sample-based, case-specific qualitative assessments were also performed.

**Figure 2:**
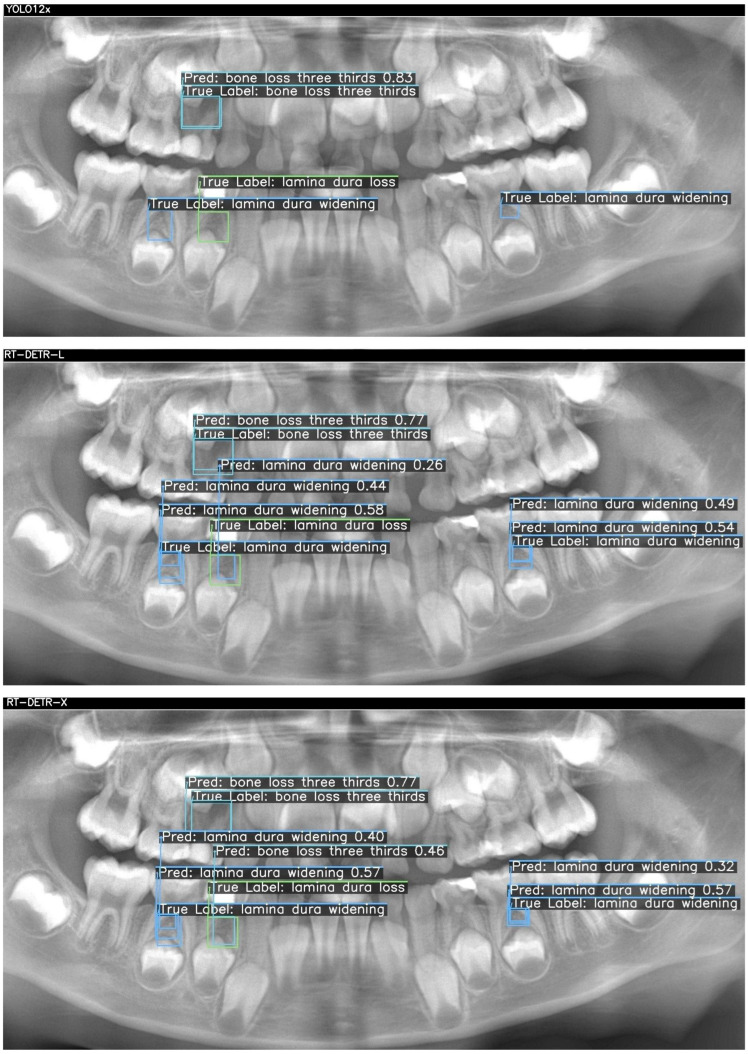
Detection results for each of the three models for a sample patient.

**Figure 3:**
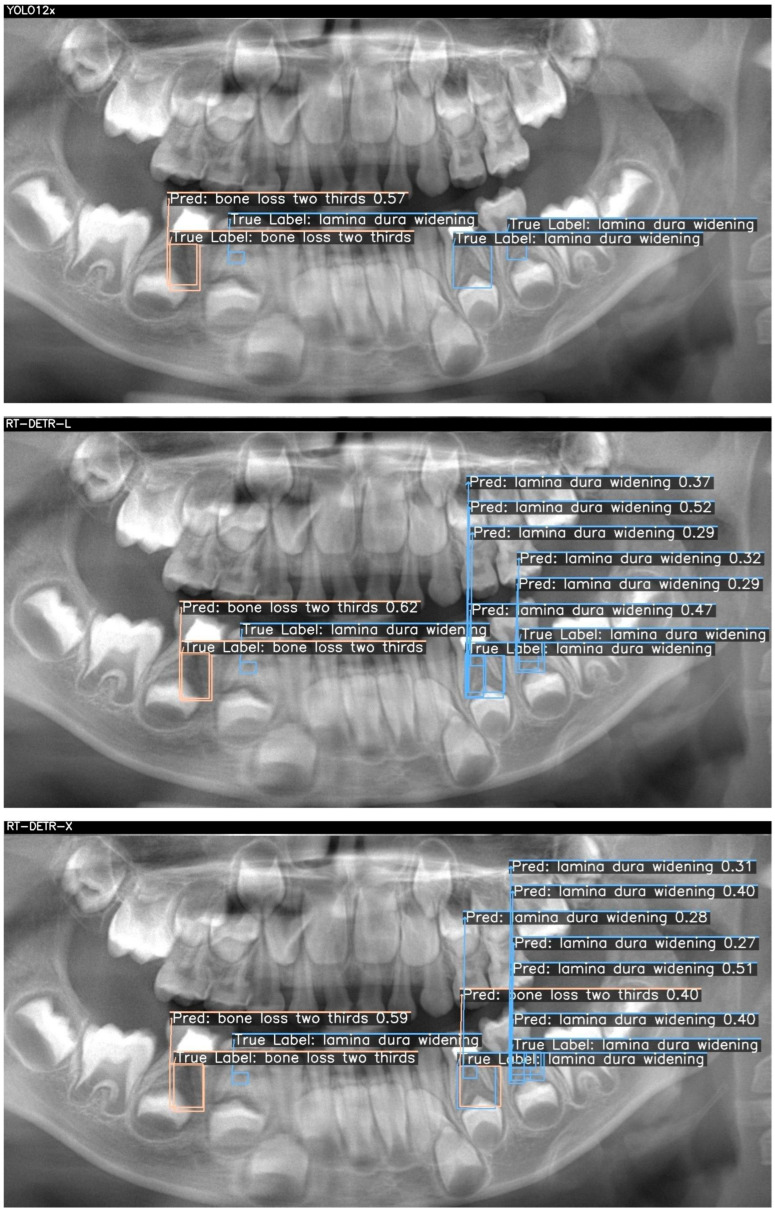
Detection results for each of the three models for another patient.

Below, two sample cases are presented as Figure 2 and Figure 3. For each case, outputs from YOLOv12x (top), RT-DETR-L (middle), and RT-DETR-X (bottom) were displayed. The "True Labels" annotated by the expert and the prediction (Pred) boxes marked by the model were color-coded in green, blue, and orange to differentiate the diseased regions during visualization.. Figure 2 and Figure 3 visualize these differences, showing that YOLOv12x frequently misses subtle lamina dura changes, RT-DETR-L captures more lesions but at the expense of false positives, while RT-DETR-X maintains superior balance by detecting both conspicuous and subtle lesions with minimal error. These visualizations further confirm the trends seen in Table 7.

In Figure 2, three ground-truth lesions were annotated on the panoramic radiograph: a central “bone loss three thirds” and two “lamina dura widening” regions in the lower molar areas. The YOLO12x model correctly detected only the central bone loss lesion (confidence = 0.83), but failed to identify either lamina dura widening (TP = 1, FN = 2, FP = 0). RT-DETR-L improved recall by capturing the bone loss (0.77) and one widening (0.44), yet introduced a spurious “lamina dura loss” prediction (TP = 2, FN = 1, FP = 1) and exhibited modest confidence scores (0.26–0.58). In contrast, RT-DETR-X achieved perfect coverage—detecting the bone loss (0.77) and both widenings (0.57, 0.32) without any false positives or negatives (TP = 3, FN = 0, FP = 0)—demonstrating superior balance between precision and recall in this challenging case as summarized in Table 7.

Figure 3 presents two true lesions: a “bone loss two thirds” in the lower left molar region and a “lamina dura widening” immediately adjacent. YOLO12x again excels at the conspicuous lesion, correctly identifying the bone loss (0.57) but missing the subtle widening (TP = 1, FN = 1, FP = 0). RT-DETR-L captures the bone loss (0.62) but overpredicts widening, generating multiple false positives (TP = 1, FN = 1, FP = 3), indicating class–label confusion despite high recall. As shown in Table 7, RT-DETR-X delivers the most reliable performance here as well, detecting both lesions (bone loss: 0.59; widening: 0.27–0.40) with only one false positive (TP = 2, FN = 0, FP = 1) and consistent confidence levels, underscoring its suitability for exhaustive lesion screening. Table 7 presents the case-level detection metrics corresponding to Figure 2 and Figure 3, confirming the numerical distribution of TP, FN, and FP values observed in the visual outputs.

The detection metrics of AI models of sample images are given in Table 7. Across both cases, RT-DETR-X consistently achieves full recall with minimal false positives, making it the most reliable for comprehensive lesion detection. YOLO12x offers rapid, high-precision identification of conspicuous bone losses but underperforms on finer lamina dura changes, suggesting a role as a first-pass screening tool. RT-DETR-L, while delivering fast inference, exhibits class confusion and a high false-positive rate, indicating its optimal use may lie in preliminary filtering rather than definitive decision support. These findings inform model selection according to clinical priorities—speed, precision, or exhaustive sensitivity—in pediatric panoramic radiograph analysis.

Since the evaluation metrics were non-inferential (precision, recall, mAP), *p*-values were not applicable. The models were compared descriptively rather than statistically, as the aim was performance benchmarking rather than hypothesis testing.

## 4. Discussion

While previous models such as those by Li et al. [3] and Tian et al. [35] primarily focused on detecting dental pathologies or localizing lesions using object detection frameworks, they lacked direct integration with treatment recommendation logic. These approaches, although technically advanced, did not extend to actionable clinical decisions, leaving a gap between detection and practice. In contrast, the present research bridges this gap by implementing a lesion-to-decision mapping strategy, directly linking radiographic severity patterns to evidence-based treatment choices—specifically, root canal treatment or extraction. This design enhances clinical applicability and moves beyond mere diagnostic support, establishing a foundation for fully integrated AI-driven decision-support systems in pediatric dentistry.

This study demonstrates that deep learning models can effectively detect periapical and furcation lesions in pediatric panoramic radiographs, supporting consistent diagnostic decision-making. Among the tested architectures, RT-DETR-X achieved the highest overall accuracy, whereas YOLOv12x provided a superior speed–performance balance, suggesting that transformer-based networks may better capture complex trabecular patterns in developing dentition. These findings are consistent with recent dental AI studies reporting improved diagnostic reproducibility through deep learning-assisted evaluation of periapical and furcation changes [5,20,27]. In the context of pediatric dentistry, such models can enhance clinical confidence and facilitate earlier intervention for progressive periapical pathology, provided that they are validated on larger and more diverse datasets.

From a clinical perspective, the taxonomy provides actionable decision support. At chairside, dentists can use predicted categories to reduce diagnostic time, improve consistency across providers, and guide referrals. This integration is expected to shorten time-to-decision, minimize variability between experienced and less experienced clinicians, and enhance workflow efficiency.

Unlike previous research focusing solely on lesion detection, this study presents a novel diagnosis-to-treatment mapping framework tailored to pediatric dentistry and this study represents the first integration of panoramic radiographic classification of primary molar furcation lesions with direct clinical decision thresholds, specifically linking lesion severity to root canal therapy or extraction protocols. By addressing this critical gap, the framework contributes to standardizing pediatric treatment planning and highlights the translational potential of AI in clinical workflows.

In practice, the workflow proceeds as follows: after a panoramic radiograph is taken, the AI system analyses the image and generates a lesion category and treatment recommendation, consistent with prior AI-assisted clinical decision-support frameworks in radiology and dentistry [9,39]. Although not conducted here, future studies should simulate clinical environments through reader studies, workflow walkthroughs, or pseudo-prospective trials to evaluate chairside usability and real-time impact. The dentist reviews these outputs in real time and incorporates them into the final clinical decision. Such workflows have been shown to reduce diagnostic delays and support less experienced clinicians in standardizing decision-making processes [21]. The novelty of the present work lies in explicitly linking lesion detection to treatment decision thresholds in pediatric molars. To our knowledge, this is the first study to integrate panoramic radiographic classification of primary molar furcation lesions with actionable treatment logic, bridging the gap between diagnostic output and clinical decision-making.

Among AI methodologies, DL, particularly CNNs, has demonstrated superior performance across several diagnostic domains, including dental caries, periodontal bone loss, vertical root fractures, and periapical lesions. Systematic reviews have reported sensitivity and specificity values for CNN-based caries detection ranging from 0.44 to 0.86 and 0.85–0.98, respectively, with AUC values typically above 0.84 [8].

In periapical diagnostics, DL models have achieved strong results even on 2D imaging. For instance, Çelik et al. [12] reported that a CNN trained on panoramic radiographs achieved an AUC of 0.91 in detecting periapical lesions in pediatric populations, demonstrating that AI tools can maintain promising diagnostic accuracy despite intrinsic limitations such as image resolution or anatomical superimposition. Similarly, Boztuna et al. [20] found precision (~0.82), recall (~0.77), and F1 scores (~0.80) using U^2^-Net models on panoramic images, underscoring the robustness of carefully trained models in periapical detection. A limitation of this study is the relatively small dataset (n = 387) from a single center, which may restrict generalizability. Future multicenter and larger-scale datasets are needed.

RT-DETR (Real-Time Detection with Transformers) models leverage attention mechanisms to simultaneously capture global image context and fine-grained local features, allowing for more accurate detection of subtle dental pathologies such as lamina dura widening or early-stage periapical radiolucency. These models decouple object queries from positional encoding, enabling flexible and precise localization even in crowded anatomical areas. Studies by Han et al. [40] and He et al. [41] have demonstrated that transformer-based detectors such as DETR and RT-DETR achieve superior performance in medical image tasks by reducing false positives and enhancing boundary delineation.

As such, the incorporation of transformer-based vision models holds substantial promise for dental radiology, where interpretability, reproducibility, and diagnostic precision are essential. The proposed framework demonstrated that transformer-based AI systems can reliably detect and classify furcation and periapical bone loss in primary molars using panoramic radiographs, linking these findings directly to clinical treatment thresholds. Among the evaluated models, RT-DETR-X achieved the highest diagnostic accuracy (mAP@0.5 = 0.43), YOLOv12x provided the most balanced trade-off between speed and precision, and RT-DETR-L offered the lowest computational demand, making it particularly suitable for low-resource or mobile clinical settings. These complementary characteristics suggest a hybrid clinical workflow, in which lightweight models perform preliminary triage followed by confirmatory assessment through higher-accuracy models or human experts [42,43].

Beyond performance metrics, this study contributes to bridging diagnostic imaging with actionable clinical decision-making in pediatric endodontics. By mapping radiographic lesion categories—such as lamina dura loss and complete furcation bone loss—to corresponding treatment recommendations (root canal therapy or extraction), the proposed AI framework establishes a clinically interpretable, reproducible, and scalable decision-support system. This aligns with the broader trend in dental AI research emphasizing lesion stratification, diagnostic standardization, and ethical implementation in real-world practice. Moreover, the reliance on panoramic radiographs rather than CBCT reinforces the method’s accessibility, affordability, and practicality—particularly valuable in pediatric or resource-constrained clinical environments.

While the results are promising, several limitations must be acknowledged. The dataset was modest (n = 387) and collected from a single center, which may restrict generalizability to broader populations and imaging conditions. Nevertheless, the dataset encompassed sufficient clinical variability to demonstrate diagnostic feasibility and proof of concept. The proposed models achieved diagnostic accuracy comparable to that reported in recent dental AI studies, underscoring their potential as reliable decision-support tools in pediatric practice. However, the absence of clinical validation through reader or inter-observer studies remains an important limitation. No inferential statistical analyses such as *p*-values or confidence intervals were performed because model evaluation was based on deterministic performance metrics including Precision, Recall, mAP@0.5 and mAP@0.5–0.95. Confidence intervals were not calculated due to the limited dataset size and the single stratified 70/20/10 split with a fixed random seed that was designed for reproducibility rather than repeated resampling, and this was considered a methodological limitation to be addressed in subsequent studies through bootstrapped confidence intervals or repeated stratified validation using larger multi-center datasets. Future research should therefore prioritize multi-reader evaluations and prospective clinical trials across multi-center, multi-ethnic cohorts to quantify the system’s contribution to diagnostic consistency among clinicians with varying experience levels. Such efforts will be essential for ensuring robust generalizability, clinical credibility, and the safe, evidence-based integration of AI-assisted diagnostic frameworks into everyday pediatric dental workflows.

## 5. Conclusions

This study is the first to integrate the panoramic radiographic classification of primary molar furcation lesions with treatment thresholds using transformer-based deep learning models. The AI framework reliably distinguished lesions requiring root canal therapy from those indicating extraction. Among the models, RT-DETR-X showed the best overall accuracy, while YOLOv12x and RT-DETR-L offered efficient and clinically practical performance. These findings highlight the potential of AI to standardize diagnosis and support evidence-based treatment decisions in pediatric dentistry.

## Figures and Tables

**Table 1:** Inclusion and exclusion criteria.

Criteria Type	Description
**Inclusion**	Radiographic detection of periapical lesions in primary molars
	Availability of high-resolution panoramic OPG images
	No history of systemic bone diseases
**Exclusion**	Severe motion artifacts or missing mandibular borders compromising image quality
	Presence of post-surgical traces
	Incomplete DICOM metadata
	Low-resolution or blurred images, missing anatomical structures, or records with artifacts not meeting international quality criteria

**Table 2:** Imaging protocol.

Parameter	Specification Details
**Device**	BLUEX Pantos DG XP Panoramic X-ray
**Power Supply**	50/60 Hz, 115–230 V
**Exposure Parameters**	60 kV, 10 mA, 12 s exposure time
**Image Format**	DICOM → Anonymization → Lossless PNG (0.1 mm/pixel)
**Quality Control**	All images reviewed for motion artifacts, missing structures, or low resolution prior to inclusion

**Table 3:** Demographic distribution.

Variable	Description	Details
**Total Number of Images**	-	387
**Age Groups**	3–6 years → Primary dentition	3–6 years (n = 120)
	7–10 years → Early mixed dentition	7–10 years (n = 140)
	11–13 years → Late mixed dentition	11–13 years (n = 127)
**Gender Distrubition**	Female/Male	48%/52%
**Clinical Relevance**	Dentition stages influence lesion visibility, prevalence, and treatment planning due to variations in furcation anatomy and root resorption.	-

**Table 4:** Ethics and data security.

Aspect	Description/Implementation
**Ethics Approval**	Approved by the Ethics Committee of Gazi University (Approval No: 2023-1264). Conducted in accordance with the Declaration of Helsinki.
**Informed Consent**	Written informed consent obtained from all participants and/or their legal guardians prior to inclusion.
**Data Source**	387 panoramic radiographs collected between 2023 and 2024 from Öveçler Oral and Dental Health Clinic, Ankara.
**Anonymization**	All patient identifiers were removed from DICOM headers, and data were stored in encrypted format.
**Data Handling Objective**	To maximize data quality and ensure patient privacy prior to model training.

**Table 5:** Radiographic categories of furcation lesions and corresponding clinical ınterpretations.

Category	Radiographic Description	Approx. Bone Loss	Clinical Interpretation
**Lamina dura widening**	Slight widening of the lamina dura or faint radiolucency confined to the furcation area	0% (no measurable bone loss)	Early inflammatory changes; possible reversible pulpitis. Lesion localized and confined to interradicular space
**Lamina dura loss**	Distinct radiolucency extending toward the apical third with partial discontinuity or irregularity of the lamina dura, but bone height largely preserved	0% (no measurable bone loss)	Initial pulpal involvement and localized inflammatory bone resorption; tooth remains restorable with pulpectomy or pulp therapy
**1/3 Bone loss**	Radiolucency extending into the furcation and involving approximately one-third of the supporting bone height. Marginal bone outline appears irregular, and trabecular pattern is disrupted	1/3–2/3 root lenght	Moderate pulpal and periodontal involvement; infection has extended into the furcation. Conservative endodontic treatment may still be feasible
**2/3 Bone loss**	Extensive furcation radiolucency reaching toward the apical third, associated with advanced destruction of lamina dura and trabecular bone	>2/3 root length	Severe bone loss and loss of structural support; pulpal necrosis and periradicular involvement likely. Extraction usually indicated
**3/3 Bone loss**	Complete loss of interradicular bone continuity with total destruction of lamina dura; root resorption or exfoliation signs may be present	3/3 root lenght	End-stage lesion or physiologic exfoliation; extraction or observation depending on eruption timing and tooth mobility

**Table 6:** Performance values of models.

AI Model	Number of Parameters	GFLOPs	Time (ms/img)	Precision	Recall	mAP@0.5	mAP@0.5–0.95
YOLO12-x	59.0 M	198.5	~17	0.442	0.333	0.397	0.163
RT-DETR-L	31.99 M	103.5	~12	0.326	0.471	0.326	0.122
RT-DETR-X	65.48 M	222.5	~24	0.440	0.483	0.434	0.187

**Table 7:** Case-level detection metrics corresponding to Figure 2 and Figure 3.

Case	Model	Ground Truth Lesions (GT)	TP	FN	FP	Figure-Based Explanation
Case 1	YOLOv12x	3 (1 bone loss + 2 widening)	1	2	0	Only bone loss detected; both widenings missed
Case 1	RT-DETR-L	3 (1 bone loss + 2 widening)	2	1	1	Bone loss + 1 widening detected; 1 widening missed; 1 false positive predicted
Case 1	RT-DETR-X	3 (1 bone loss + 2 widening)	3	0	0	All ground-truth lesions detected; no errors
Case 2	YOLOv12x	2 (1 bone loss + 1 widening)	1	1	0	Bone loss detected; widening missed
Case 2	RT-DETR-L	2 (1 bone loss + 1 widening)	1	1	3	Bone loss detected; widening missed; 3 false positives generated
Case 2	RT-DETR-X	2 (1 bone loss + 1 widening)	2	0	1	Both ground-truth lesions detected; 1 false positive prediction

## Data Availability

Due to patient privacy and ethical restrictions, the datasets generated and/or analyzed during the current study are not publicly available but are available from the corresponding author on reasonable request and with permission of the institutional ethics committee.
